# Sudden Cardiac Death: An Update on Commotio Cordis

**DOI:** 10.7759/cureus.38087

**Published:** 2023-04-24

**Authors:** Ovie Okorare, Gabriel Alugba, Soremi Olusiji, Endurance O Evbayekha, Akanimo U Antia, Emmanuel Daniel, Daniel Ubokudum, Olanrewaju K Adabale, Anderson Ariaga

**Affiliations:** 1 Internal Medicine, Nuvance Health Vassar Brothers Medical Center, New York, USA; 2 Internal Medicine, Delta State University, Abraka, NGA; 3 Internal Medicine, New York Medical College, Metropolitan Hospital Center, New York, USA; 4 Internal Medicine, St. Luke's Hospital, Chesterfield, USA; 5 Medicine, Lincoln Medical and Mental Health Center, New York, USA; 6 Internal Medicine, Trinity Health System, Livonia, USA; 7 Internal Medicine, Thomas Hospital, Fairhope, USA; 8 Internal Medicine, East Carolina University, Greenville, USA

**Keywords:** young athletes, blunt impact, ventricular fibrillation, commotio cordis, sudden cardiac death (scd)

## Abstract

Sudden cardiac death (SCD) is one of the leading causes of cardiovascular mortality, and it is caused by a diverse array of conditions. Among these is commotio cordis, a relatively infrequent but still significant cause, often seen in young athletes involved in competitive or recreational sports. It is known to be caused by blunt trauma to the chest wall resulting in life-threatening arrhythmia (typically ventricular fibrillation). The current understanding pertains to blunt trauma to the precordium, with an outcome depending on factors such as the type of stimulus, the force of impact, the qualities of the projectile (shape, size, and density), the site of impact, and the timing of impact in relation to the cardiac cycle.

In the management of commotio cordis, a history of preceding blunt chest trauma is usually encountered. Imaging is mostly unremarkable except for ECG, which may show malignant ventricular arrhythmias. Treatment is focused on emergent resuscitation with the advanced cardiac life support protocol algorithm, with extensive workup following the return of spontaneous circulation. In the absence of underlying cardiovascular pathologies, implantable cardiac defibrillator insertion is not beneficial, and patients can even resume physical activity if the workup is unremarkable. Proper follow-up is also key in the management and monitoring of re-entrant ventricular arrhythmias, which are amenable to ablative therapy. Prevention of this condition involves protecting the chest wall against blunt trauma, especially with the use of safety balls and chest protectors in certain high-risk sporting activities.

This study aims to elucidate the current epidemiology and clinical management of SCD with a particular focus on a rarely explored etiology, commotio cordis.

## Introduction and background

Sudden cardiac death (SCD) is one of the leading causes of cardiovascular mortality, and it is defined as an unexpected death from cardiac arrest occurring within an hour of symptom onset if the onset is witnessed or within one day of having been well and symptom-free if unwitnessed [1]. The major mechanism linked to this occurrence is a cardiac arrhythmia (as seen in rare cases of commotio cordis), hypertrophic cardiomyopathies, congenital anomalies, and coronary artery disease (CAD) [2,3].

A meta-analysis has shown smoking and diabetes to be major risk factors while having an implantable cardioverter defibrillator (ICD) was noted to be a protective factor against SCD [4]. A rare but noteworthy cause of SCD, commotio cordis, defined as an event in which blunt trauma not penetrating the chest leading to fatal cardiac arrhythmia (the most common type being ventricular fibrillation) in the absence of cardiac damage, has received much-needed attention over the past few years. It is more prevalent in males, and has an estimated incidence of 10-20 cases annually [5,6], making it the second leading cause of SCD in young athletes in the United States [7]. An analysis of the US Commotio Cordis Registry revealed a 95% male predominance [8]. The male preponderance has been postulated to be related to the participation of males in sports in which commotio cordis occurs, but it is also speculated that there may be some sex-related as well as genetic susceptibility, as the number of women participating in competitive sports has increased without a change in the condition's incidence among them [9]. The mechanisms of injury include motor vehicle accidents, traumatic falls, explosions, and projectile impacts during sporting activities such as baseball, football, and lacrosse [10].

## Review

Methodology 

We based our study on a defined set of inclusion and exclusion criteria.

Inclusion Criteria

We only included studies published within the last 10 years. We searched PubMed, Google Scholar, and the Cochrane databases for relevant studies. We included articles from 2013 to 2023 and considered systematic reviews, meta-analyses, randomized control trials, and clinical trials. Keywords for the search included “sudden death” and “commotio cordis”; we combined the keywords in every combination to generate all possible articles for screening. Our keyword combinations and search results generated a total of 400 articles. We read the abstracts with our objectives, the inclusion criteria, and the exclusion criteria (below) in mind, which narrowed them down to 60 full-text articles. Ultimately, we included 46 articles in our review.

Exclusion Criteria

We excluded all studies published before 2013, as well as case reports, case series, articles in languages other than English, and non-full-text articles.

Epidemiology of sudden cardiac death

Cardiovascular disease remains the leading cause of death globally, accounting for approximately 17.8 million deaths worldwide in 2017 [11]. SCD accounts for approximately 40-50% of all cardiovascular deaths and 15-20% of all deaths [12], most of which are due to cardiac arrest from ventricular tachyarrhythmias. In the United States, approximately 350,000 people die yearly from SCD [13].

The World Health Organization defines SCD as sudden unexpected death within one hour of symptom onset or within 24 hours of having been last seen well [4]. Cardiac arrest usually presents without any warning signs or symptoms and is usually fatal [14,15]. When the death is not witnessed, the “sudden” period extends to 24 hours. SCD can be the first presentation of cardiovascular disease, and almost half of all SCD victims have no previously diagnosed heart condition [16]. In the past two decades, cardiovascular-related deaths have become less common in developed countries, especially in individuals with risk factors for SCD such as CAD and heart failure (HF). However, recent studies from the United States still show an increasing incidence of cardiac arrest, with over 350,000 cases occurring outside healthcare facilities [17] and 290,000 in-hospital annually [18].

Risk factors for SDC are not well established compared to those for ischemic heart disease and stroke. Some suspected or established risk factors for SDC include overweight and obesity, diabetes mellitus, hypertension, arrhythmias, cigarette smoking, CAD, and male sex [19]. Physical activity is an established protective factor against CAD, cerebrovascular disease, and heart failure, even though the association between physical activity and SCD has not been extensively studied [19].

Commotio cordis

Commotio cordis is defined as the mechanical stimulation of the heart by nonpenetrating impulse-like impact to the precordium that, through intrinsic cardiac mechanisms, gives rise to disturbances of the cardiac rhythm of varying type, duration, and severity, including SCD, in the absence of structural damage that would explain any observed effects [20]. This phenomenon arises due to sudden high-energy, blunt impact to the chest wall, evoking fatal arrhythmia resulting in SCD. Ventricular fibrillation is known to be the most frequent arrhythmia associated with commotio cordis, but studies have also reported polymorphic ventricular tachycardia, heart block, and atrial fibrillation [9,21].

This uncommon mechano-electric event leading to fatal arrhythmia occurs commonly in young males involved in sporting activities [22]. This could be a result of the fact that younger males are more commonly involved in compact sports, and younger individuals have higher chest compliance than older adults [23]. Commotio cordis was found to be the second leading cause of sudden death in young males, after hypertrophic cardiomyopathy [7]. Although it occurs commonly during competitive or recreational sporting activities (especially in sports associated with high-velocity moving projectiles, e.g., baseball, ice hockey, lacrosse, karate, and cricket), commotio cordis has also been seen in nonsporting activities such as traffic accidents, violent attacks, children playing, or corporal punishment [24-26]. Baseball has been reported to be the leading cause of commotio cordis in the US [27], with 7.3% of baseball injuries being commotio cordis [28]. Another study has reported that 3% of football fatalities at the high school and college levels were commotio cordis [29].

The physical impact in all scenarios of commotio cordis is directly on the left precordium over the cardiac silhouette. Considering the nature of physical sports, the occurrence of blunt chest trauma is common, but the incidence of commotio cordis is quite low. In the event of commotio cordis, the risk of mortality is very high if cardiopulmonary resuscitation (CPR) and defibrillation are not begun promptly [8]. Even with this intervention, patients who regain spontaneous circulation are prone to neurological disability [30].

Commotio cordis causes approximately 20% of SCD in athletes in the United States [7]. It usually manifests clinically as cardiovascular collapse, which is mostly instantaneous, although it has been reported that nearly 20% of patients remain conscious for some brief moments after the impact [5]. Some patients may develop urinary incontinence or dyspnea within moments of impact [31]. Commotio cordis appears to occur more frequently in thin individuals with a compliant chest wall [5].

Mechanism

The key mechanism of commotio cordis is a blunt traumatic impact on the heart resulting in the occurrence of malignant arrhythmia. Kohl et al. [32] proposed different factors that could affect the severity of the arrhythmias seen in commotio cordis. Firstly, the type of mechanical stimulus involved: smaller, more compact objects that could cause a higher risk of generating mechanically induced arrhythmia than larger objects that distribute their energy over a wider surface area. Second, the force of impact, linked to the velocity, size, shape, and hardness of the impacting object [5,8]. Studies have revealed that velocities of approximately 64 km/hour are likely to cause commotio cordis [33].

Third, the site of impact must be directly over the heart, at the cardiac silhouette, for ventricular fibrillation. Studies have shown that the impact directly over the center of the left ventricle is more lethal, causing ventricular fibrillation, than those on the periphery of the heart, which is not associated with ventricular fibrillation [34]. Finally, the timing of the impact: in relation to the cardiac cycle, the impact must occur during the ventricular depolarization during the upslope of the T-wave. This has been considered the vulnerable period of the cardiac cycle [35]. This results in rapid elevation of left ventricular intracavitary pressure after the impact triggers stretch-activated ion channels (stretch-sensitive ion channels, such as ATP-dependent K+ [10], K+Ach, calcium, sodium, and other potassium channels), which subsequently develop into focal ventricular depolarization amidst repolarization, resulting in ventricular fibrillation [36].

Clinical approach toward patients with suspected commotio cordis

Diagnosis

The most important factor is witnessed blunt chest trauma in relatively young and healthy patients. The trauma is usually immediately followed by collapse. If available, an ECG may reveal ventricular fibrillation. This is important because the diagnosis of commotio cordis cannot be made in the presence of underlying heart disease or severe traumatic injury causing gross myocardial injury [6]. The characteristics of chest blows vary but usually involve heavy objects traveling with projectile force (lacrosse ball, baseball, hockey puck) that hit the chest directly or nearly over the heart. The presence of protective gear does not exclude the diagnosis of commotio cordis, as more than a third of patients who suffer from commotio cordis wear adequate protective gear [37].

Treatment

Emergent resuscitation is the cornerstone of treatment following the identification of commotio cordis. Basic and advanced life support resuscitation algorithms should be instituted immediately. Adequate CPR, airway management, and prompt defibrillation should be given as indicated [6]. Following the return of spontaneous circulation (ROSC), a comprehensive evaluation including clinical examination, ECG, genetic testing for channelopathies (e.g., Brugada syndrome, long QT syndrome), and imaging studies is appropriate. In the absence of any underlying cardiovascular pathology such as hypertrophic cardiomyopathy, long QT syndromes, or ion channel disorders, ICDs offer no benefit in preventing future similar episodes [6]. The American College of Cardiology/American Heart Association (ACC/AHA) released a consensus statement on the disqualifications and eligibility of athletes who suffer an episode of commotio cordis. They recommend that the athlete resume training if the evaluation for cardiovascular pathology is negative. This recommendation was partially due to the rarity of commotio cordis occurring more than once in healthy individuals and the lack of data to judge the predisposition to commotio cordis [6].

Prevention

Commotio cordis can be prevented by avoiding a hit to the chest wall. It may also be prevented by using chest protection and softballs in sports like baseball, hockey, and lacrosse. It is important to train young athletes in techniques to avoid a hit to the chest wall, such as turning quickly from blunt objects. While protective clothing is encouraged to prevent commotio cordis, data from the US Commotio Cordis Register did not show a statistically significant difference in survival between those who wore and those who did not wear chest padding [38]. A systematic review that examined chest protection in experimental models using animals and biomechanical models overall has suggested that current evidence does not suggest reliable protection from the use of chest protectors, but a reduction in ventricular fibrillation as the speed of the ball increased was noticed [39]. With increased awareness of commotio cordis, sports equipment manufacturers have been working on more robust designs for chest protectors. It is unclear if this would be effective in preventing commotio cordis [23].

Experimental studies on animals using the Tufts experimental model of commotio cordis used chest protectors made with a combination of Accelleron, Airilon foam, Tridur, and ImpacShield. This was found to reduce the incidence of ventricular fibrillation from 54% to 5% when compared to controls [40]. Safety balls have been well studied in comparison with standard baseballs and are generally recommended. Safety baseballs have been shown to reduce the risk of commotio cordis in experimental models. As demonstrated in a systematic review of experimental studies, the softest balls led to the lowest incidence of ventricular fibrillation induction [39]. Resuscitation is key to preventing deaths from commotio cordis, and the availability of defibrillators at sporting events should be ensured. Improvements in the rate of survival related to commotio cordis have been noted over time, with survival rates now approaching 60%. Data from the US Commotio Cordis Registry showed that of 16 cases in which automated external defibrillators (AEDs) were used, 11 survived, as opposed to only 49 of 200 persons surviving without the use of AEDs [38]. There was a statistically significant benefit from having an onsite defibrillator and from a time-to-resuscitation of less than three minutes [38]. Young athletes and coaches should be trained in effective CPR.

## Conclusions

Commotio cordis is an established but rare cause of SCD and is seen primarily in young athletes following blunt trauma to the precordium from a projectile such as a lacrosse ball, baseball, and even a hit from a fist. Factors such as the velocity and force of impact, impact site, size of the object, and timing of the impact in relation to the cardiac cycle are associated with the occurrence of commotio cordis. Prompt initiation of CPR and defibrillation is known to improve outcomes in patients. Preventive measures such as the use of chest wall protectors and safety balls have been studied, and they have been recommended in certain sports.
